# 

**Published:** 1878-04-20

**Authors:** 


☞ All communications relating to the business of THE RECORD, for the years 1877 and 1878, must be addressed to DR. R. C. WORD, Managing Editor Southern Medical Record, Atlanta, Ga.

☞ Brief and practical communications are solicited on all subjects pertaining to medicine; also reports of cases in practice.

☞ Send money by check, postal order or registered letter.

☞ Write your name, post-office, county and State plainly.

Receipted 1878.—Drs. A. L. M. Hawkins, J. R. Wilson, John H. Henry, M. T. Bell, W. H. Wells, T. A. Cook, J. S. Milling, J. M. Stansill, F. Courtney, F. DeLee, J. H. Reynolds, R. R. Ibrie, J. W. Bennett, R. H. Davis, J. S. Carothers, J. T. McDouell, T. J. Blackamore, J. M. Lewis, M. Demaret, W. G. Noyes, T. J. Brasher, J. S. L. Miller, J. E. Martin, W. H. Whitehead, M. J. Luster, E. N. Cushing, J. D. Terrell, P. M. Catching, E. Y. Flemming, W. S. Posey;—F. M. Canton, 1877 and 1878, R. D. Lucius, 1877; W, J. Judd, 1876 and 1877.

